# Characteristics of serum neurofilament light chain as a biomarker in hereditary spastic paraplegia type 4

**DOI:** 10.1002/acn3.51518

**Published:** 2022-02-16

**Authors:** Christoph Kessler, Lina Maria Serna‐Higuita, Carlo Wilke, Tim W. Rattay, Holger Hengel, Jennifer Reichbauer, Elke Stransky, Alejandra Leyva‐Gutiérrez, David Mengel, Matthis Synofzik, Ludger Schöls, Peter Martus, Rebecca Schüle

**Affiliations:** ^1^ Department of Neurodegenerative Diseases, Hertie Institute for Clinical Brain Research and Center of Neurology University of Tübingen Tübingen Germany; ^2^ German Center for Neurodegenerative Diseases (DZNE) Tübingen Germany; ^3^ Department of Clinical Epidemiology and Applied Biostatistics University of Tübingen Tübingen Germany; ^4^ Center of Neurology University of Tübingen Tübingen Germany

## Abstract

**Objective:**

While the anticipated rise of disease‐modifying therapies calls for reliable trial outcome parameters, fluid biomarkers are lacking in spastic paraplegia type 4 (SPG4), the most prevalent form of hereditary spastic paraplegia. We therefore investigated serum neurofilament light chain (sNfL) as a potential therapy response, diagnostic, monitoring, and prognostic biomarker in SPG4.

Methods: We assessed sNfL levels in 93 patients with SPG4 and 60 healthy controls. The longitudinal study of sNfL levels in SPG4 patients covered a baseline, 1‐year follow‐up and 2‐year follow‐up visit.

**Results:**

Levels of sNfL were significantly increased in patients with genetically confirmed SPG4 compared to healthy controls matched in age and sex (*p* = 0.013, *r* = 0.2). Our cross‐sectional analysis revealed a greater difference in sNfL levels between patients and controls in younger ages with decreasing fold change of patient sNfL elevation at older ages. Over our observational period of 2 years, sNfL levels remained stable in SPG4 patients. Disease severity and progression did not correlate with sNfL levels.

Interpretation: Our longitudinal data indicate a stable turnover of sNfL in manifest SPG4; therefore, sNfL levels are not suitable to monitor disease progression in SPG4. However, sNfL may be valuable as a therapy response biomarker, since its turnover could be modified by interventions. As the course of sNfL levels appears to be most dynamic around the onset of SPG4, the ability to detect a therapy response appears to be especially promising in younger patients, matching the need to initiate treatment in early disease stages.

## Introduction

Hereditary spastic paraplegia (HSP) constitutes a heterogeneous group of genetic diseases characterized by slowly progressive spastic paraparesis. The most common genotype is autosomal‐dominantly transmitted SPG4 caused by mutations of the *SPAST* gene. SPG4 accounts for 17%–32% of all^1^, ^2^ and 31%–61% of autosomal dominant HSP cases.^1^, ^3^, ^4^ Classified as a pure HSP phenotype according to Harding's criteria,^5^ symptoms in SPG4 are typically restricted to spastic paraparesis, sphincter disturbances, and mild dorsal column sensory impairment. As of today, the diagnostic workup relies on neurological examination, genetic testing, MRI imaging, and neurophysiology, while progression is evaluated mostly using clinical outcomes. Easily accessible fluid biomarkers are missing in SPG4 for both the clinical as well as the research context. Neurofilament light chain (NfL) is a promising candidate biomarker for both purposes, as it is elevated to different degrees across various neurological diseases^6^ and mirrors the degeneration of corticospinal tract fibers.^7^ Serum NfL (sNfL) has been shown to be a diagnostic and prognostic biomarker in amyotrophic lateral sclerosis (ALS),^8^, ^9^ the most common motor neuron disease, and multiple sclerosis (MS),^10^ a possible mimic of HSP. Yet, data on sNfL levels in SPG4 are missing. The only previous study investigating sNfL in HSP found levels to be significantly increased compared to controls, but neither specified sNfL levels in genetically defined subgroups such as SPG4 nor provided data on the influence of demographic factors.^11^ However, as NfL levels physiologically increase with age and are influenced by sex,^12^, ^13^ these aspects must be considered. Moreover, potential contexts of use for sNfL in HSP, for example, as a potential therapy response, diagnostic, prognostic or monitoring biomarker, were never explored.^14^ Here, we comprehensively investigate sNfL levels in SPG4 and provide the first data on the longitudinal course of a molecular biomarker in SPG4.

## Subjects and methods

### Subjects

A total of 153 participants (93 SPG4 patients, 60 controls) were recruited from the Department of Neurology, Hertie Institute for Clinical Brain Research, University Hospital Tübingen. All patients were examined by a neurologist with special expertise in HSP and diagnosed with SPG4 based on genetic testing. Truncating mutations (nonsense/stop‐gain mutations, splice site mutations, exon deletions, frame‐shift insertions, or deletions) of the SPAST gene were found in 71 patients (76.3%) and missense mutations (including in‐frame insertions or deletions) in 19 patients (20.4%). The exact mutation was unknown in three patients (3.2%). Disease severity was assessed by the spastic paraplegia rating scale (SPRS),^15^ with baseline scores being available for 87 patients. Further demographic data are detailed in Table 1. The control cohort consisted of healthy volunteers, comprising 10 subjects (5 females, 5 males) for each decade between 20 and 80 years of age, resulting in a total of 60 controls. All controls were examined by a neurologist with special expertise in neurodegenerative diseases. Median and mean age were similar in patients and controls (median age in patients 52.0 years, mean 50.9, range 11.7–82.1; median age in controls 49.6, mean 49.3, range 21.7–77.2; two‐sided Mann–Whitney test, *p* = 0.632). While age was evenly distributed across decades in controls, a majority of patients were between 40 and 59 years old (Supplementary Figure S1, Supplementary Table S2). The distribution of sex was equal across patients and controls (male/female ratio 1.1 in patients and 1.0 in controls, two‐sided chi‐square test, *p* = 0.745). In a first model for the longitudinal analysis of sNfL levels in patients, we chose periods of 7–18 months (“1‐year follow‐up”) and 19–30 months (“2‐year follow‐up”) from baseline. All baseline visits (*n* = 93) including those of patients without follow‐up visits were incorporated in the analysis. If patients had multiple visits within a follow‐up period, the last available visit was selected. We thereby selected a total of 45 follow‐up visits, including 31 data points for the 1‐year follow‐up and 14 data points for the 2‐year follow up. Existence of a 1‐year follow‐up was not mandatory to be included in the 2‐year follow‐up cohort (only 1‐year follow up: *n* = 22; only 2‐year follow‐up: *n* = 5; 1‐year and 2‐year follow‐up: *n* = 9). The median time from baseline to the 1‐year follow up was 13 months (mean 13.0, range 7–18), and 25 months (mean 24.7, range 21–29) to the 2‐year follow‐up, respectively. In a second model, we shortened the time windows for the 1‐year and the 2‐year follow‐up to 9–15 months and 21–27 months after the baseline visit, respectively. This resulted in 39 follow‐up visits, including 25 for the 1‐year follow‐up and 14 for the 2‐year follow‐up. In this model, the median time from baseline to the 1‐year follow‐up was 12.0 months (mean 12.6, range 10–15), and 24.5 months (mean 24.7, range 21–26) to the 2‐year follow‐up, respectively. To capture marked disease progression as defined by a practically relevant change in ambulation at the 1‐year follow‐up, we used the SPATAX disability scale, ranging from 0 (no disability) to 7 (confined to bed).^16^

**Table 1:** Demographics and serum neurofilament light chain (sNfL) levels of patients and controls.

	SPG4	Controls
Sample size	93	60
Age (years)	52.0 (44.2–58.4)	49.6 (32.0–64.4)
Sex (male/female ratio)	1.1	1.0
Age of onset (years)	36.0 (21–44.5)	NA
Disease duration (years)	15.6 (8.0–26.4)	NA
Disease severity (SPRS score)	19 (12–26)	NA
Average slope of SPRS score (gained points per year)	0.9 (0.0–2.0)	NA
Mutation status (missense/truncating/unknown)	20.4% / 76.3% / 3.2%	NA
sNfL (pg/ml)	12.4 (9.1–16.7)	10.2 (6.0–16.1)
Annual increase in sNfL (pg/ml)	2.3%	3.0%

Values of age, age of onset, disease duration, disease severity, average slope of the SPRS score and sNfL levels are detailed as medians and interquartile ranges. Missense mutations: missense mutations, in‐frame insertions or deletions. Truncating mutations: nonsense/stop‐gain mutations, splice site mutations, exon deletions, frame‐shift insertions or deletions.

For the investigation of sNfL as a prognostic biomarker, we calculated longitudinal clinical disease progression rates based on the SPRS. For this analysis, we selected patients with at least one follow‐up visit within a time interval of 6 months to 4 years after baseline. If more than one follow‐up visit was available, the last visit within the specified follow‐up interval was selected. This resulted in a cohort of 43 patients with a total of 86 visits and a median time to follow‐up of 1.9 years (mean 2.0, range 0.9–4.0).

Informed written consent was obtained from participants or their legal representatives. The local ethics committee approved the study (172/2018BO2, 199/2011BO1).

### Biomaterial

Serum samples were frozen at −80°C within 60 min after collection, stored in the local biobank of the Hertie Institute for Clinical Brain Research and analyzed without previous thaw–freeze cycles. None of the samples were collected after physical exercise.

### NfL measurements

Serum NfL concentrations were assessed in duplicates by single molecule array (Simoa) technology as previously described^17^ with a dilution factor of 1:4. Reference samples were analyzed in quadruplicates. The inter‐assay percent coefficients of variation (CV) were 14.1%, 13.6%, and 11.6% for reference samples with mean concentrations of 9.8 pg./mL, 45.2 pg./mL, and 120.6 pg./mL, respectively. The intra‐assay CVs were 3.1%‐3.3%.

### Statistical analysis

Categorical variables are described using relative and absolute frequencies, numerical variables are reported as either mean (± standard deviation) or median (interquartile range), depending on the distribution of the data. Normality of the distribution was assessed by investigating kurtosis, skewness, as well as by visual inspection of the frequency distribution (histogram), boxplot, P–P plot (probability‐probability plot), and Q‐Q plot (quantile‐quantile plot).

We used nonparametric methods to analyze group effects for data without log‐transformation. The effect size was calculated as $r=¦\frac{Z}{\sqrt{n}}¦,$ where z is the z‐score and n is the number of observations.^18^ To simultaneously control for the influence of multiple factors on sNfL levels, we performed a two‐way ANCOVA analysis with log‐transformed sNfL concentrations in order to meet the assumption of normality. To examine the value of sNfL as a prognostic biomarker, a linear regression model was carried out, including sNfL at baseline, age, and the annualized change of the SPRS score (SPRS score at follow‐up minus SPRS score at baseline, divided by the time to follow‐up in years). In order to assess the temporal dynamics of sNfL based on cross‐sectional data, we matched each control to one patient, controlling for age and sex (propensity score matching in the mode “optimal matching”, according to Thoemmes, F. (2012). Propensity score matching in SPSS. arXiv:1201.6385). To assess the balance of covariate distribution between treatment groups, the standardized mean difference (SMD) was calculated. We thus established a subgroup of 60 patients and 60 controls. We then calculated the sNfL ratio of each matched pair and studied the course of sNfL ratios by the mean age of matched pairs. The mean age in matched SPG4 patients was 50.1 years (SD ± 14.4) and the mean age in controls was 49.3 years (SD ± 17.2) (SMD = 0.05). The distribution of sex was also equal across patients and controls (male/female ratio 1.1 in patients and 1.0 in controls) (SMD = 0.07).

The analysis of longitudinal sNfL measurements was conducted using a linear mixed model allowing for missing data points, as the number of follow‐up visits differed between patients (see *Subjects and Methods* section). The covariates of the model were selected by minimizing the Akaike information criterion (AIC). To model the temporal course of sNfL, we calculated predicted sNfL levels at baseline as $\text{sNfL}=10^{0.616+\mathrm{Age}\;\mathrm{at}\;\text{baseline}\times 0.01}$ (0.616 = intercept, 0.01 = coefficient of the ANCOVA of log‐transformed sNfL levels in SPG4; see *Results* section) and plotted the results in Figure 2B. All procedures were carried out using IBM SPSS statistics software version 25. Graphs were created in SAS JMP version 14.

## Results

### Serum NfL levels are elevated in SPG4 patients

**Figure 1:**
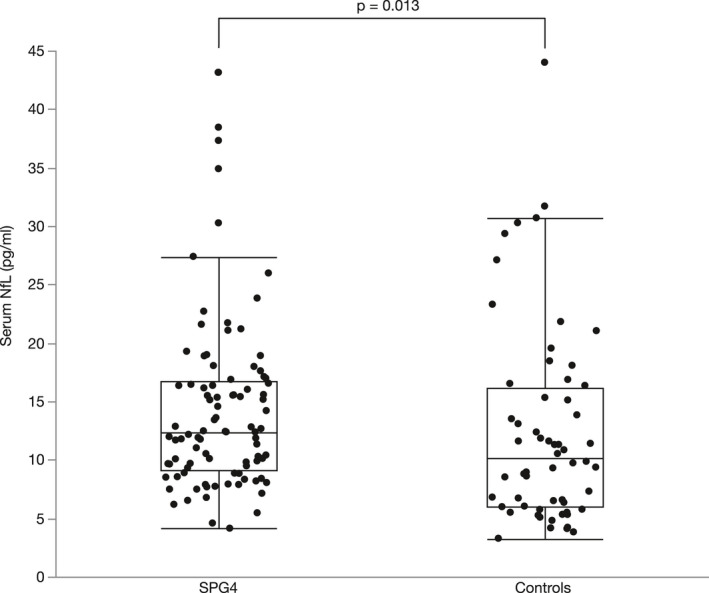
Scatter and box plot of serum NfL levels in SPG4 and controls. Horizontal lines represent medians, boxes show interquartile ranges, and whiskers extend to the outermost data points within 1.5 interquartile ranges.

SPG4 patients had a median sNfL level of 12.4 pg./ml at baseline (IQR: 9.1–16.7), being significantly higher than in age‐ and sex‐matched controls with a median 10.2 pg./ml (IQR: 6.0–16.1; two‐sided Mann–Whitney test, *p* = 0.013, *r* = 0.2; Fig. 1). This finding was confirmed by two‐way ANCOVA, controlling for the influence of age and sex (*p* = 0.003, F(1, 149) = 9.3, B = 0.078, R^2^ = 0.59). According to our model, sNfL levels were increased by 20% in patients compared to controls as calculated by back‐transformation of the log‐level coefficient B. Assessing the sNfL levels in 60 matched pairs of patients and controls (see *Statistical analysis* section and next paragraph) also yielded a significant elevation of sNfL levels in patients (median 12.6 pg./ml, IQR 9.9–17.7; two‐sided Wilcoxon test, *p* = 0.005, *r* = 0.36).

### Age influences serum NfL levels in SPG4 patients and controls

**Figure 2:**
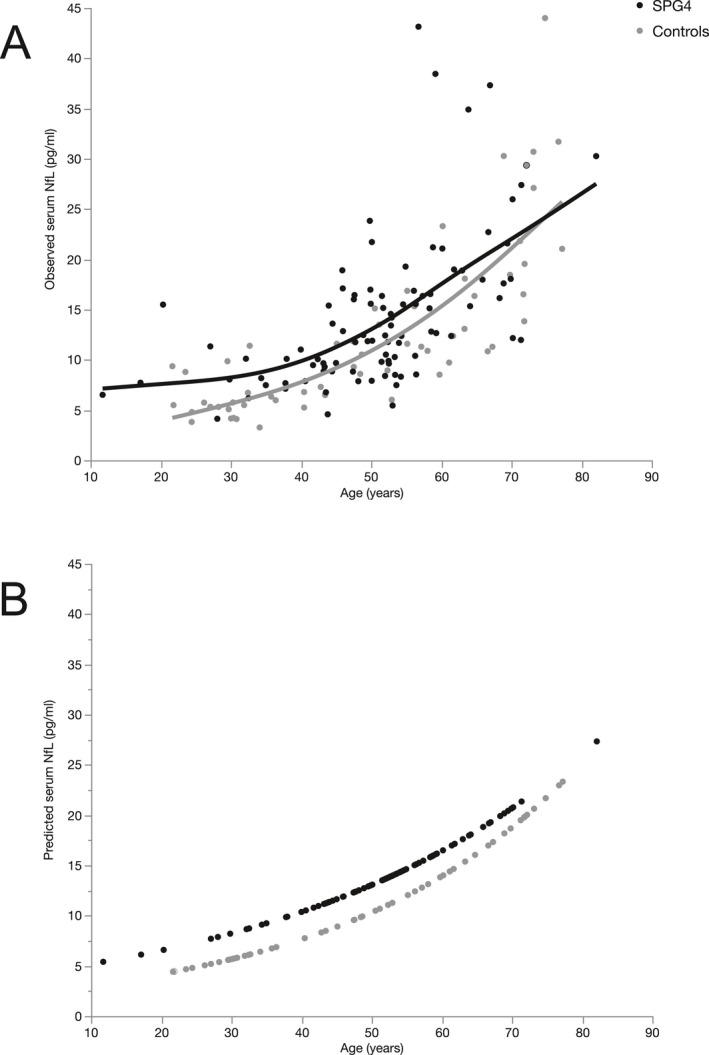
Scatter plot of serum NfL levels by age in SPG4 (black dots) and controls (grey dots). (A) Observed values fitted with cubic splines (lambda = 7.5). (B) Values predicted by an exponential model (see *Statistical analysis* section).

**Figure 3:**
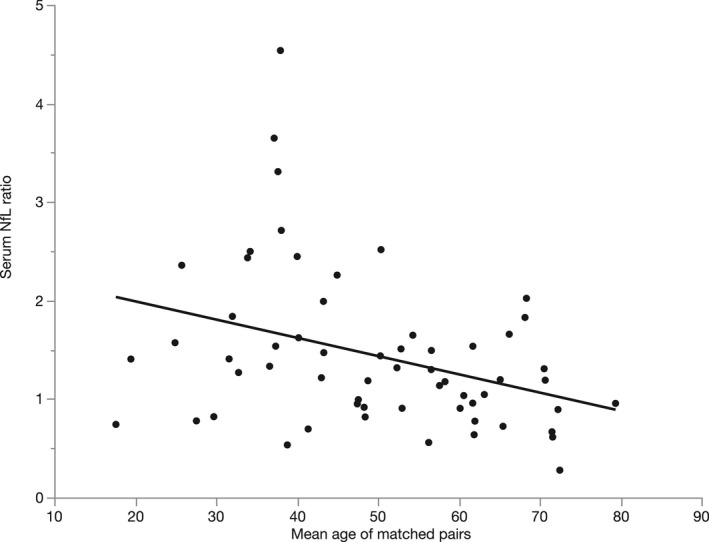
SPG4 patients' individual serum NfL ratios, calculated by dividing the serum NfL levels of 60 patients by the serum NfL levels of their matched controls, with linear fit.

**Figure 4:**
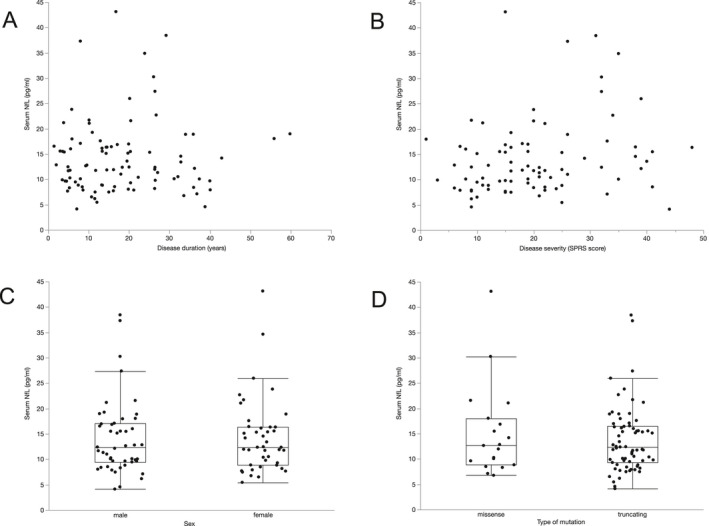
Disease duration (A), disease severity (B), sex (C) and the type of mutation (D) lack a significant influence on serum NfL levels. The statistical analysis was performed controlling for age.

To further investigate which factors influence sNfL levels, we conducted separate ANCOVA analyses of cross‐sectional data sets for patients and controls. For patients, we included age, sex, mutation status (truncating vs. missense), disease duration, disease severity, and their interactions in the model. Using backward selection, only age was found to have a significant impact on sNfL levels, leading to an annual increase of 2.3% in SPG4 patients (*p* < 0.001, F(1, 91) = 54.0, B = 0.01, R^2^ = 0.372). In controls, age, and sex were evaluated. Similar to HSP cases, age was also a significant factor in controls with an annual increase of 3.0% (*p* < 0.001, F(1, 59) = 143.9, B = 0.013, R^2^ = 0.713), while sex did not significantly influence sNfL levels. Since the age‐related increase was higher in controls and graphical exploration of sNfL levels by age suggested a smaller increase in sNfL levels in SPG4 patients compared to controls in older subjects (Fig. 2), we sought to examine the relationship between patients' and controls' sNfL levels over time. We therefore performed a one‐to‐one matching of patients and controls as detailed in the *Statistical analysis* section, leading to a subgroup of 60 patients and controls matched pairwise in age and sex. In this subgroup, we calculated the sNfL ratio of each matched pair (patient/control). This ratio significantly decreased with increasing mean age of matched pairs (Spearman's ρ = −0.397, *p* = 0.002; Fig. 3), showing that patients' fold increases in sNfL compared to matched controls decline in older subjects. Factors without a significant effect on sNfL levels in SPG4 are shown in Figure 4.

### The performance of sNfL as a diagnostic biomarker is age‐dependent

**Figure 5:**
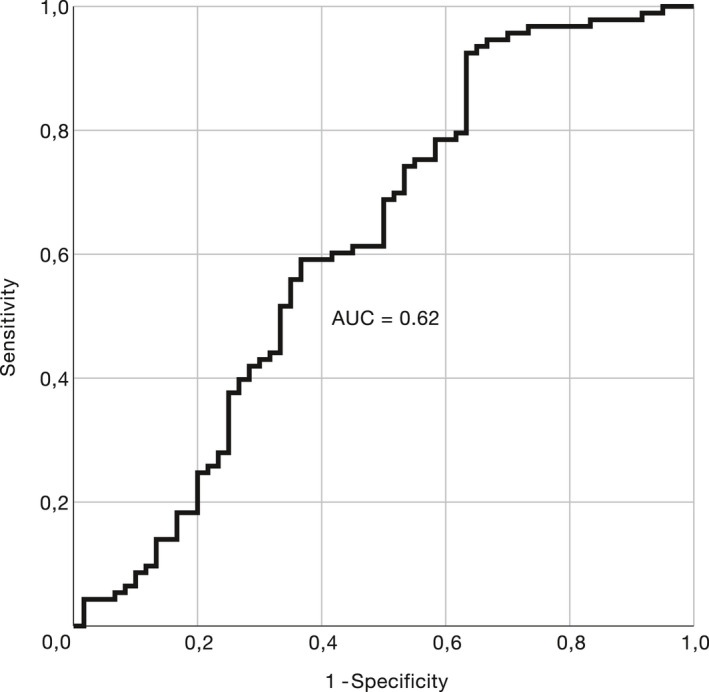
Performance of serum NfL in separating SPG4 patients from controls (ROC analysis, 95% CI: 0.52–0.72, *p* = 0.013).

To assess the use of serum NfL as a diagnostic biomarker, we conducted ROC analyses for all subjects and for different age groups, as the fold increase of sNfL as measured by the sNfL ratio was smaller in older patients. Considering all participants, sNfL performed modestly in discriminating patients from controls (AUC = 0.62, 95% CI: 0.52–0.72, *p* = 0.013; Fig. 5). While not being valuable in the group ≥60 years (AUC 0.52, 95% CI: 0.33–0.70, *p* = 0.861), the performance of sNfL was moderately good in subjects <60 years (AUC 0.77, 95% CI: 0.68–0.86, *p* < 0.001).

### Serum NfL levels do not predict the clinical course of SPG4

**Figure 6:**
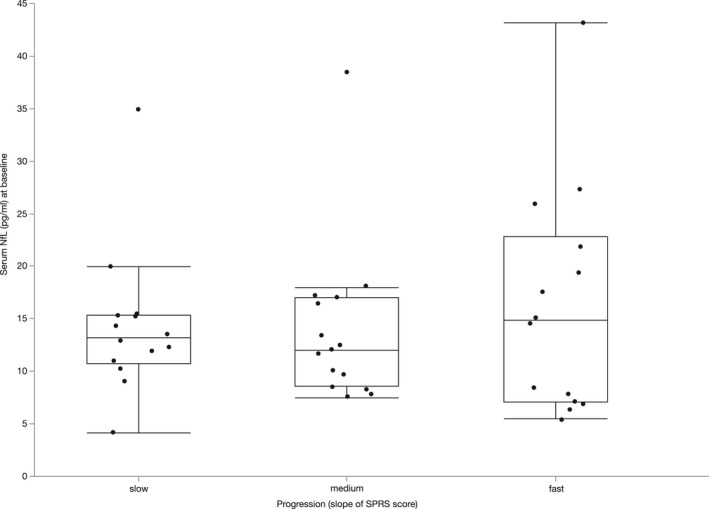
Serum NfL levels at baseline do not predict disease progression as measured by individual slopes of the SPRS score. Groups (slow/medium/fast progression) were established for graphical illustration by tercile split of SPRS score slopes. Horizontal lines represent medians, boxes show interquartile ranges, and whiskers extend to the outermost data points within 1.5 interquartile ranges. The statistical analysis was performed controlling for age (age‐adjusted sNfL ratio; see *Statistical analysis* section).

To examine the clinical course of SPG4, we calculated the individual annualized change of the SPRS score from baseline to the last follow‐up visit within a period of 6 months to 4 years in 43 patients. As age influences sNfL levels in SPG4 (see above), a linear regression model was carried out, including sNfL at baseline, age, and the annualized change of the SPRS score. We did not find a significant correlation between the annualized change of the SPRS score and sNfL levels (*p* = 0.90, F(2, 42) = 0.10, R^2^ = 0.005) or the annualized changes of subscores comprising the first six (*p* = 0.65, F(2, 42) = 0.44, R^2^ = 0.02) and 10 items (*p* = 0.64, F(2, 42) = 0.51, R^2^ = 0.03) of the SPRS score, respectively (Fig. 6). Therefore, sNfL is not suited as a prognostic biomarker in manifest SPG4 within 6 months to 4 years from baseline.

### Longitudinal course of sNfL levels in SPG4

**Figure 7:**
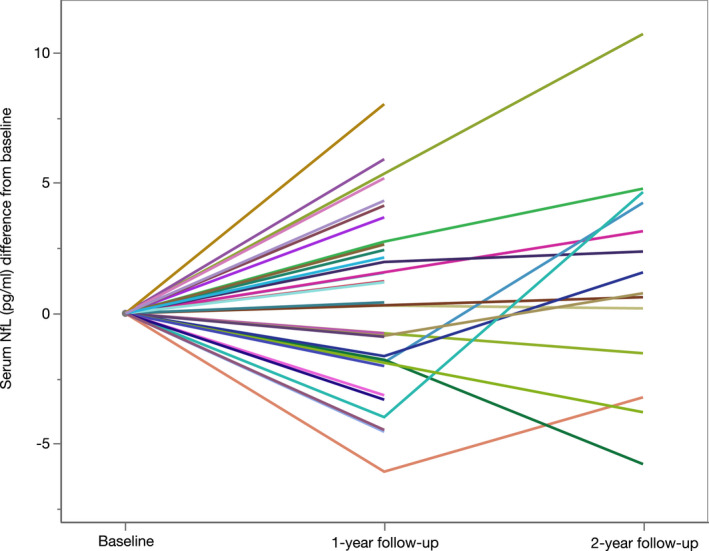
Intraindividual course of serum NfL levels in SPG4 displayed as the change from baseline levels. One‐year follow‐up: 7–18 months from baseline; two‐year follow‐up: 19–30 months from baseline. Each colored line represents one patient. [Colour figure can be viewed at wileyonlinelibrary.com]

We used a linear mixed model to assess the intraindividual course of sNfL levels in SPG4 patients with follow‐up visits and age at baseline as covariates. As detailed in the section on statistical analysis, in a first model visits were grouped into baseline, 1‐year follow‐up (7–18 months past baseline) and 2‐year follow‐up (19–30 months past baseline) visits. Levels of sNfL did not change significantly over time (*p* = 0.103, Fig. 7). Like in the ANCOVA restricted to cross‐sectional data, only age at baseline was found to have a significant influence on sNfL levels (*p* < 0.001, F(1, 93.4) = 59.0, B = 0.01). The effect of age on sNfL levels was comparable to our observation in the cross‐sectional data set. At the 1‐year follow‐up, three of 31 patients showed a relevant worsening of ambulation as defined by a change in the SPATAX disability scale. Levels of sNfL were not markedly elevated in these patients. In a second model, we narrowed the periods to 9–15 months and 21–27 months after baseline for the 1‐year and 2‐year follow‐up, respectively. Like in the first model, sNfL did not change significantly over time (*p* = 0.166).

### CSF NfL levels in SPG4

Levels of cNfL were available for six patients that were part of a previous study on cNfL in a mixed HSP cohort.^19^ In these patients, cNfL levels ranged from 681 pg./ml to 1952 pg./ml with a median of 991 pg./ml. Serum NfL levels ranged from 9.6 pg./ml to 38.4 pg./ml with a median of 14.1 pg./ml. Levels of cNfL are shown in Supplementary Figure S3.

## Discussion

We here present the first data on sNfL levels in SPG4, the most prevalent subtype of HSP. We found sNfL levels to be significantly elevated in patients compared to age‐ and sex‐matched controls, consistent with the neurodegenerative nature of the disease. As sNfL levels were 20% higher in patients compared to controls, the relative increase in SPG4 is similar to a previously published mixed HSP cohort^11^ and Friedreich's ataxia,^20^ but lower than in Alzheimer's disease (AD),^21^ Parkinson's disease (PD),^22^ and spinocerebellar ataxia type 3 (SCA3)^23^ as further examples of slowly progressive neurodegenerative diseases. In rapidly progressive neurodegenerative diseases like ALS^24^, ^25^ and Creutzfeldt–Jakob disease (CJD),^26^ markedly higher sNfL levels have been reported compared to our findings in SPG4. Therefore, sNfL could support the clinically relevant differential diagnosis of SPG4 and other motor neuron diseases like ALS, as it has been discussed for mixed HSP cohorts.^11^, ^19^ When exploring the discriminatory power of sNfL in SPG4 patients and controls, our analysis yielded a moderate performance, concurring with the comparatively low increase of sNfL in SPG4, thus limiting the usefulness in this scenario. The role of sNfL as a diagnostic biomarker in differentiating SPG4 from disease mimics like primary lateral sclerosis (PLS) and primary progressive MS (PPMS) remains to be demonstrated by direct comparisons. Considering the subtypes of HSP, it is unknown to which degree pathways leading to sNfL release differ. In SPG4, focal axonal swellings are a common finding,^27^, ^28^ possibly caused by impaired microtubule severing and decreased axonal transport.^29^ While each subtype of HSP has its distinct genetic cause and pathophysiology, length‐dependent axonal degeneration has been consistently found in pathologic studies,^30^ and imaging studies have reported spinal cord atrophy and corticospinal tract abnormalities in a variety of HSP subtypes.^31^, ^32^, ^33^ We therefore believe axonal degeneration and the subsequent release of NfL to be a common downstream process caused by possibly distinct upstream pathways. Nevertheless, differences in the rate of axonal decay and the involvement of additional neuroanatomical structures may lead to different sNfL levels across the subtypes of HSP.

In patients with SPG4, only age proofed to have a significant influence on sNfL levels, while sNfL did not depend on sex, disease severity, disease duration, and mutation status (missense vs. truncating). Remarkably, the annual age‐related increase was lower in patients (2.3%) than in controls (3.0%). The different age‐related sNfL increases in patients and controls indicate a closing gap between sNfL levels of patients and controls in older subjects (Fig. 2). The relative increase of sNfL in SPG4 patients compared to controls is thus larger at younger age and earlier disease stages (Fig. 3), potentially indicating higher NfL release in early disease stages. Similar temporal dynamics of NfL have been described in a mixed HSP cohort,^19^ Alzheimers's disease,^34^ SCA3,^23^ and Friedreich's ataxia,^20^ suggesting a distinct pattern in these slowly progressive neurodegenerative diseases.

The missing impact of disease severity and duration on sNfL levels in SPG4 (see above) highlights that sNfL levels are independent of the current state of SPG4, but are likely determined by a function involving (i) the rate of axonal decay and (ii) substrate quantity. Since axons––in particular those of the corticospinal tract––are the substrate of degeneration in SPG4, reduction of the axonal compartment over the course of the disease may prevent a significant rise in sNfL levels in later disease stages. The rate of axonal decay cannot be determined by our study, but could be assumed to be steady given the mostly linear and slowly progressive clinical course of SPG4. When comparing our findings to results obtained by other methods, imaging studies of the spinal cord and the corticospinal tract seem most suited, as they provide cross‐sectional data on the course of SPG4. Several magnetic resonance imaging studies in patients with SPG4 have found atrophy of the spinal cord^31^, ^32^ or abnormalities of the corticospinal tract.^33^, ^35^, ^36^, ^37^ Some of these studies have reported a correlation of disease duration and severity with the extent of corticospinal tract abnormalities on the other side.^36^, ^37^ This is in line with our hypothesis that the vanishing of long tract axons leads to a less pronounced sNfL increase in later disease stages due to decreasing substrate quantity. In Friedreich's ataxia, spinal cord atrophy has been shown to correlate with age and disease duration^38^; in SCA3, a correlation with disease duration and severity has been demonstrated.^39^ While these studies are methodically and statistically different from our investigation, they illustrate the continuing damage to long tract axons and may therefore explain the similar temporal dynamics of sNfL in these slowly progressive diseases. In more rapidly progressive ALS, declining sNfL levels in later stages have been described in cross‐sectional analyses^8^, ^40^ and hypothesized to be a result of a decrease in substrate quantity.^41^ However, longitudinal investigations have yielded stable sNfL levels in ALS, so the cross‐sectional findings must be interpreted cautiously.^8^, ^24^, ^40^ In SPG4 and other slowly progressive neurodegenerative diseases, the effect of slow disease‐related axonal loss is probably weighed out by the rising age‐related release of sNfL, altogether leading to a comparatively low increase in sNfL levels in aging patients. In controls, the age‐related increase in sNfL levels is higher than in patients and particularly steep in patients above 60 years (Supplementary Figure S1). The steeper increase in older ages has been reported by several authors and may be caused by subclinical neurological diseases, but also be part of normal aging; cutoff values to differentiate these two processes have not been established.^42^, ^43^

While our findings suggest a significant increase in sNfL levels before, at or after disease onset, the exact timing of the sNfL increase in relation to disease onset needs to be examined by longitudinal assessments of presymptomatic mutation carriers. Elevated sNfL levels have been found in this group across several neurodegenerative diseases.^23^, ^24^, ^34^, ^44^, ^45^ If sNfL was also elevated in presymptomatic SPG4 mutation carriers, specifying the onset of sNfL elevation could help to define the optimal start of a disease‐modifying therapy, as an increase could serve as a marker of commencing axonal degeneration before clinical disease onset. However, increased sNfL levels in presymptomatic carriers have not yet been demonstrated in SPG4 or other forms of HSP and therefore remain subject to further research.

In manifest SPG4, we did not find sNfL to be a prognostic biomarker of the progression rate, as sNfL levels could not predict the change in the SPRS score in a follow‐up of approximately 2 years. This differs from findings in ALS, where higher sNfL levels at baseline are associated with worse prognosis.^46^ Our results also do not support the use of sNfL as a monitoring biomarker in SPG4, as disease severity and duration lacked a significant influence on sNfL levels in our cross‐sectional data. In addition, the longitudinal assessment of individual sNfL levels with follow‐up visits approximately 1 and 2 years after baseline did not yield a significant change. The adynamic intraindividual course of sNfL mirrors our findings in the cross‐sectional analysis, where the age‐related increase in sNfL levels was lower in SPG4 patients than in controls. However, the missing suitability of sNfL as a monitoring biomarker does not rule out its use as a parameter of therapy response in upcoming clinical trials. While a monitoring biomarker is expected to change with alterations of patients' clinical status, a therapy‐response biomarker should display a biological response following a treatment and is therefore not tied to being a monitoring biomarker.^14^ For SPG4, this seems especially promising in younger patients, as the relative increase in sNfL is larger than in older patients and sNfL levels may therefore be more susceptible to therapeutic interventions. Although the low temporal dynamics of sNfL in SPG4 may lead to small effect sizes, requiring large sample sizes in clinical trials, there are precedents that NfL can be used as a therapy response biomarker even if NfL levels do not rise with progressing disease. In relapsing–remitting MS, sNfL levels have been shown to decrease significantly depending on the type of pharmacotherapy, thus establishing sNfL as a therapeutic response biomarker.^47^, ^48^, ^49^ More importantly, cNfL levels decline following treatment with Nusinersen in spinal muscular atrophy type 3 (SMA3), a disease also exhibiting a slowly progressive course. Levels of cNfL decreased in a clinical trial of Tofersen in ALS while not correlating with clinical measures; therefore, cNfL levels indicated a therapy response without being a monitoring biomarker over the length of the trial.^50^ Thus, the use of sNfL as a therapy‐response biomarker in SPG4 needs to be examined in clinical trials.

In conclusion, we demonstrate sNfL levels are increased in patients with SPG4. The magnitude of the sNfL elevation in patients compared to controls was equal to or lower than in other slowly progressive neurodegenerative diseases. Levels of sNfL increased with age, but showed distinct temporal dynamics indicating a marked rise in younger patients and relatively static levels afterwards. Given the comparatively larger increase of sNfL levels in younger patients, longitudinal assessments in presymptomatic mutation carriers are warranted to examine if sNfL levels start to rise before symptom onset. Considering sNfL as a diagnostic biomarker, our results point to a robust performance of sNfL in differentiating SPG4 from more aggressive motor neuron diseases like ALS. However, we did not find evidence that sNfL could serve as a prognostic or monitoring biomarker in SPG4. The use of sNfL as a therapy‐response biomarker in SPG4 remains to be examined in clinical trials.

Limitations of our study include a selection bias of our cohort toward adult onset cases and age at examination of about 40 to 59 years.^1^, ^4^, ^16^ This is presumably due to the role of our institution as a tertiary referral center for adult patients. Another limitation is the comparatively small number of follow‐up visits for the longitudinal analysis of sNfL levels, especially for the two‐year follow‐up. Furthermore, the levels and course of sNfL in presymptomatic SPG4 mutation carriers are unknown and need to be investigated.

## Author Contributions

CK: Design and conceptualization of the study, acquisition of data (patient recruitment, patient assessment, serum sampling), analysis of data, drafting, and revision of the manuscript. LMSH: Analysis of data, revision of the manuscript. CW: Acquisition of data (patient recruitment, patient assessment, serum sampling), revision of the manuscript. TWR: Acquisition of data (patient recruitment, patient assessment, serum sampling), revision of the manuscript. HH: Acquisition of data (patient recruitment, patient assessment, serum sampling), revision of the manuscript. JR: NfL measurements, revision of the manuscript. ES: NfL measurements, revision of the manuscript. AL: NfL measurements, revision of the manuscript. MS: Revision of the manuscript. DM: Technical advice, revision of the manuscript. LS: Acquisition of data (patient recruitment, patient assessment, serum sampling), revision of the manuscript. PM: Analysis of data, revision of the manuscript. RS: Design and conceptualization of the study, acquisition of data (patient recruitment, patient assessment, serum sampling), drafting, and revision of the manuscript.

## Conflict of Interest

Dr. Schüle reports grants from National Institute of Neurological Disorders and Stroke, grants from Bundesministerium für Bildung und Forschung (BMBF), grants from European Union's Horizon 2020 research and innovation program and grants from HSP Research Foundation, during the conduct of the study. All other authors have nothing to disclose.

## Supporting information


**Supplementary Table S1** Clinical characteristics of patients with SPG4 at baseline visits.

**Supplementary Table S2** Age at examination of patients and controls by decade.

**Supplementary Table S3** Age at onset of patients by decade.

**Supplementary Figure S1** Levels of sNfL in patients and controls by decades. Horizontal lines represent medians, boxes show interquartile ranges, and whiskers extend to the outermost data points within 1.5 interquartile ranges. Boxplots are shown for subgroups with at least 10 subjects.

**Supplementary Figure S2** Ratio of sNfL levels in 60 patients and matched controls. The horizontal line represents the median, the box shows the interquartile range, and whiskers extend to the outermost data points within 1.5 interquartile ranges.

**Supplementary Figure S3** Levels of CSF and serum NfL in six patients with SPG4.
