# Supplementary material for: Characteristics of serum neurofilament light chain as a biomarker in hereditary spastic paraplegia type 4
Source: Ann Clin Transl Neurol. 2022 Feb 16;9(3):326–38. doi: 10.1002/acn3.51518 (PMC8935322; doi:10.1002/acn3.51518)
Supplement: Supplementary file 1 — Supplementary Table S1 Clinical characteristics of patients with SPG4 at baseline visits. [file ACN3-9-326-s006.docx]

**Supplementary Table 1: Clinical characteristics of patients with SPG4 at baseline visits**

| **#** | **Age at examination** | **Age at onset** | **Sex** | **Type of mutation** | **SPRS score** | **Cognitive status** |
| --- | --- | --- | --- | --- | --- | --- |
| 1 | 12 | 1 | female | truncating | 10 | normal |
| 2 | 17 | 1 | female | truncating | 9 | normal |
| 3 | 20 | 0 | male | missense | 41 | mild cognitive impairment |
| 4 | 27 | 0 | male | truncating | 21 | normal |
| 5 | 28 | 21 | male | truncating | 44 | normal |
| 6 | 30 | 10 | male | truncating | 12 | normal |
| 7 | 32 | 1 | male | truncating | 19 | normal |
| 8 | 33 | 21 | male | truncating | 9 | normal |
| 9 | 34 | 8 | female | truncating | 24 | normal |
| 10 | 35 | 20 | female | truncating | 15 | normal |
| 11 | 38 | 33 | female | truncating | 15 | normal |
| 12 | 38 | 1 | male | missense | 33 | normal |
| 13 | 38 | 30 | male | truncating | 8 | normal |
| 14 | 40 | 22 | male | truncating | 26 | normal |
| 15 | 41 | 32 | male | truncating | 7 | normal |
| 16 | 42 | 35 | male | truncating | 10 | normal |
| 17 | 42 | 5 | male | missense | 35 | normal |
| 18 | 43 | 39 | male | truncating | 16 | normal |
| 19 | 43 | 30 | female | truncating | 11 | normal |
| 20 | 43 | 23 | female | truncating | 19 | normal |
| 21 | 44 | 10 | female | missense | 21 | normal |
| 22 | 44 | 5 | male | truncating | 9 | normal |
| 23 | 44 | 40 | female | truncating | 15 | normal |
| 24 | 44 | 37 | female | missense | 12 | normal |
| 25 | 44 | 25 | female | unknown | 40 | normal |
| 26 | 45 | 5 | male | truncating | 14 | normal |
| 27 | 46 | 10 | female | truncating | 26 | normal |
| 28 | 46 | 38 | male | truncating | 18 | normal |
| 29 | 46 | 44 | male | missense | 6 | normal |
| 30 | 47 | 34 | male | truncating | 25 | normal |
| 31 | 48 | 42 | male | truncating | 8 | normal |
| 32 | 48 | 32 | female | truncating | 38 | normal |
| 33 | 48 | 36 | female | truncating | 20 | normal |
| 34 | 48 | 27 | male | unknown | 22 | normal |
| 35 | 49 | 44 | female | truncating | 9 | normal |
| 36 | 49 | 35 | female | truncating | 18 | normal |
| 37 | 50 | 44 | female | truncating | 20 | normal |
| 38 | 50 | 30 | male | truncating | 19 | normal |
| 39 | 50 | 47 | female | truncating | 19 | normal |
| **#** | **Age at examination** | **Age at onset** | **Sex** | **Type of mutation** | **SPRS score** | **Cognitive status** |
| 40 | 50 | 10 | female | truncating | 9 | normal |
| 41 | 50 | 40 | female | truncating | 9 | normal |
| 42 | 50 | 34 | female | truncating | 18 | normal |
| 43 | 51 | 25 | female | truncating | 15 | normal |
| 44 | 52 | 37 | female | truncating | 16 | normal |
| 45 | 52 | 32 | male | truncating | not available | normal |
| 46 | 52 | 16 | male | truncating | 20 | normal |
| 47 | 52 | 32 | male | truncating | 32 | normal |
| 48 | 52 | 20 | female | truncating | 21 | normal |
| 49 | 52 | 47 | female | truncating | 22 | normal |
| 50 | 52 | 49 | male | truncating | 3 | normal |
| 51 | 53 | 48 | male | missense | not available | normal |
| 52 | 53 | 20 | female | truncating | 38 | normal |
| 53 | 53 | 20 | female | truncating | 16 | normal |
| 54 | 53 | 10 | female | missense | 29 | normal |
| 55 | 53 | 41 | female | truncating | 25 | normal |
| 56 | 53 | 48 | male | missense | 11 | normal |
| 57 | 53 | 45 | female | missense | 22 | normal |
| 58 | 54 | 42 | male | truncating | 16 | normal |
| 59 | 54 | 49 | male | truncating | 16 | normal |
| 60 | 54 | 49 | male | missense | 6 | normal |
| 61 | 54 | 36 | female | truncating | 21 | normal |
| 62 | 55 | 51 | male | truncating | 14 | normal |
| 63 | 55 | 44 | male | truncating | 16 | normal |
| 64 | 56 | 39 | male | missense | 15 | normal |
| 65 | 56 | 34 | female | truncating | 23 | normal |
| 66 | 56 | 43 | male | truncating | 19 | normal |
| 67 | 56 | 40 | male | truncating | 41 | normal |
| 68 | 57 | 40 | female | missense | 15 | normal |
| 69 | 57 | 43 | female | truncating | 48 | normal |
| 70 | 58 | 44 | female | truncating | 10 | normal |
| 71 | 58 | 57 | male | truncating | 7 | normal |
| 72 | 59 | 49 | male | truncating | 12 | normal |
| 73 | 59 | 55 | male | truncating | 11 | normal |
| 74 | 59 | 30 | male | truncating | 31 | normal |
| 75 | 59 | 50 | male | missense | 19 | normal |
| 76 | 60 | 50 | female | missense | 22 | normal |
| 77 | 61 | 35 | female | truncating | 21 | normal |
| 78 | 62 | 2 | male | truncating | not available | normal |
| 79 | 63 | 29 | male | truncating | not available | normal |
| **#** | **Age at examination** | **Age at onset** | **Sex** | **Type of mutation** | **SPRS score** | **Cognitive status** |
| 80 | 64 | 40 | female | unknown | 35 | normal |
| 81 | 64 | 39 | female | truncating | 25 | normal |
| 82 | 66 | 60 | male | truncating | 1 | normal |
| 83 | 67 | 40 | female | truncating | 34 | normal |
| 84 | 67 | 59 | male | truncating | 26 | normal |
| 85 | 68 | 55 | female | truncating | not available | normal |
| 86 | 69 | 56 | female | truncating | 33 | normal |
| 87 | 69 | 49 | male | missense | 20 | normal |
| 88 | 70 | 14 | male | missense | not available | normal |
| 89 | 70 | 50 | female | truncating | 39 | normal |
| 90 | 70 | 34 | male | truncating | 39 | normal |
| 81 | 71 | 45 | female | missense | 25 | normal |
| 92 | 71 | 45 | male | truncating | 32 | normal |
| 93 | 82 | 56 | male | missense | 32 | normal |

Age is given in years.
