# Supplementary material for: Characteristics of serum neurofilament light chain as a biomarker in hereditary spastic paraplegia type 4
Source: Ann Clin Transl Neurol. 2022 Feb 16;9(3):326–38. doi: 10.1002/acn3.51518 (PMC8935322; doi:10.1002/acn3.51518)
Supplement: Supplementary file 2 — Supplementary Table S2 Age at examination of patients and controls by decade. [file ACN3-9-326-s005.docx]

**Supplementary Table 2: Age at examination of patients and controls by decade**

| **Decade (age in years)** | **Number of patients** | **Number of controls** |
| --- | --- | --- |
| 10-19 | 2 | 0 |
| 20-29 | 4 | 10 |
| 30-39 | 8 | 10 |
| 40-49 | 25 | 10 |
| 50-59 | 36 | 10 |
| 60-69 | 13 | 10 |
| 70-79 | 4 | 10 |
| 80-89 | 1 | 0 |
