# Supplementary material for: Characteristics of serum neurofilament light chain as a biomarker in hereditary spastic paraplegia type 4
Source: Ann Clin Transl Neurol. 2022 Feb 16;9(3):326–38. doi: 10.1002/acn3.51518 (PMC8935322; doi:10.1002/acn3.51518)
Supplement: Supplementary file 3 — Supplementary Table S3 Age at onset of patients by decade. [file ACN3-9-326-s003.docx]

**Supplementary Table 3: Age at onset of patients by decade**

| **Decade (age in years)** | **Number of patients** |
| --- | --- |
| 0-9 | 11 |
| 10-19 | 7 |
| 20-29 | 12 |
| 30-39 | 24 |
| 40-49 | 28 |
| 50-59 | 10 |
| 60-69 | 1 |
