# Supplementary material for: Characteristics of serum neurofilament light chain as a biomarker in hereditary spastic paraplegia type 4
Source: Ann Clin Transl Neurol. 2022 Feb 16;9(3):326–38. doi: 10.1002/acn3.51518 (PMC8935322; doi:10.1002/acn3.51518)
Supplement: Supplementary file 4 — Supplementary Figure S1 Levels of sNfL in patients and controls by decades. Horizontal lines represent medians, boxes show interquartile ranges, and whiskers extend to the outermost data points within 1.5 interquartile ranges. Boxplots are shown for subgroups with at least 10 subjects. [file ACN3-9-326-s002.docx]

**Supplementary Figure 1:** Levels of sNfL in patients and controls by decades. Horizontal lines represent medians, boxes show interquartile ranges, and whiskers extend to the outermost data points within 1.5 interquartile ranges. Boxplots are shown for subgroups with at least ten subjects.
