# Supplementary material for: Characteristics of serum neurofilament light chain as a biomarker in hereditary spastic paraplegia type 4
Source: Ann Clin Transl Neurol. 2022 Feb 16;9(3):326–38. doi: 10.1002/acn3.51518 (PMC8935322; doi:10.1002/acn3.51518)
Supplement: Supplementary file 5 — Supplementary Figure S2 Ratio of sNfL levels in 60 patients and matched controls. The horizontal line represents the median, the box shows the interquartile range, and whiskers extend to the outermost data points within 1.5 interquartile ranges. [file ACN3-9-326-s001.docx]

**Supplementary Figure 2:** Ratio of sNfL levels in 60 patients and matched controls. The horizontal line represents the median, the box shows the interquartile range, and whiskers extend to the outermost data points within 1.5 interquartile ranges.
