# Supplementary material for: Characteristics of serum neurofilament light chain as a biomarker in hereditary spastic paraplegia type 4
Source: Ann Clin Transl Neurol. 2022 Feb 16;9(3):326–38. doi: 10.1002/acn3.51518 (PMC8935322; doi:10.1002/acn3.51518)
Supplement: Supplementary file 6 — Supplementary Figure S3 Levels of CSF and serum NfL in six patients with SPG4. [file ACN3-9-326-s004.docx]

**Supplementary Figure 3:** Levels of CSF and serum NfL in six patients with SPG4.
